# How Tasmanian Emergency Departments ‘Choose Wisely’ When Investigating Suspected Pulmonary Embolism

**DOI:** 10.3390/healthcare11111599

**Published:** 2023-05-30

**Authors:** Lauren E. Thurlow, Pieter J. Van Dam, Sarah J. Prior, Viet Tran

**Affiliations:** 1 Tasmanian School of Medicine, College of Health and Medicine, University of Tasmania, Hobart, TAS 7000, Australia; 2 School of Nursing, College of Health and Medicine, University of Tasmania, Burnie, TAS 7320, Australia; 3 Tasmanian School of Medicine, College of Health and Medicine, University of Tasmania, Burnie, TAS 7320, Australia; 4 Emergency Department, Royal Hobart Hospital, Hobart, TAS 7000, Australia; 5 Menzies Institute for Medical Research, University of Tasmania, Hobart, TAS 7000, Australia

**Keywords:** computed tomography angiography, emergency service, hospital, medical overuse, pulmonary embolism, clinical practice guidelines, quality improvement

## Abstract

Overuse of computed tomography pulmonary angiograms (CTPAs) for diagnosis of pulmonary embolism (PE) has been recognised as an issue for over ten years, with Choosing Wisely Australia recommending that CTPAs only be ordered if indicated by a clinical practice guideline (CPG). This study aimed to explore the use of evidence-based practice within regional Tasmanian emergency departments in relation to CTPA orders by determining whether CTPAs were ordered in accordance with validated CPGs. We conducted a retrospective medical record review of all patients who underwent CTPA across all public emergency departments in Tasmania between 1 August 2018 and 31 December 2019 inclusive. Data from 2758 CTPAs across four emergency departments were included. PE was reported in 343 (12.4%) of CTPAs conducted, with yield ranging from 8.2% to 16.1% between the four sites. Overall, 52.1% of participants had neither a CPG documented, nor a D-dimer conducted before their scan. A CPG was documented before 11.8% of scans, while D-dimer was conducted before 43% of CTPAs. The findings presented in this study indicate that Tasmanian emergency departments are not consistently ‘Choosing Wisely’ when investigating PE. Further research is required to identify explanations for these findings.

## 1. Introduction

In Australia in 2009, almost 10,000 people were diagnosed with acute pulmonary embolism (PE), with PE increasing as our population ages [1]. If untreated, acute PE is associated with a mortality rate as high as 30% [2]. In addition, the clinical presentation of acute PE is non-specific, making diagnosis difficult [3]. Computed tomography pulmonary angiograms (CTPAs) provide rapid and reliable results and have become the primary technique for diagnosing or excluding a PE [4].

Overuse of this imaging type has been recognised as an issue for over ten years, with Choosing Wisely Australia [5], in conjunction with The Australasian College for Emergency Medicine and The Royal Australian and New Zealand College of Radiologists, recommending that imaging for suspected PE is not to be requested, unless indicated by a clinical practice guideline (CPG), such as the Wells Score [6], or Charlotte Rule [7]. They also specify that PE should be ruled out in low-risk patients identified through CPGs using the PE Rule Out Criteria [8], followed by a D-dimer if indicated, not CTPA. The PE Rule Out Criteria cannot be used in patients aged 50 and over. Therefore, a D-dimer must be conducted in this age group. D-dimer levels increase with age, and as a result, the age-adjusted D-dimer cut-off was developed to assist in ruling out PE in those aged 50 years or older [9], without needing to proceed to image.

Concerns about CTPA overuse have primarily come from data from American emergency departments, where the yield can be as low as 5% [10]. While there is some debate around the ‘ideal’ CTPA yield, the Royal College of Radiologists in the United Kingdom specifies that PE should be evident in at least 15% of CTPAs, with alternative diagnoses evident in at least a further 50% of the scans conducted [11]. Mountain and Keijzers [4] supported this nominated yield, using the Colleges’ recommendation in their 2016 multi-centre review on CTPA use in Australasia.

CTPA overuse is linked to increased healthcare costs [12], exposure to ionising radiation leading to increased lifetime malignancy risk [13], extravasation of contrast, and anaphylaxis [14]. The authors conducted a literature review and identified that CTPAs continue to be overused in emergency departments and that while CPGs have a strong effect on reducing unnecessary CTPAs, with no significant increased risk in clinically significant PEs, the adoption of these tools by emergency department clinicians has remained low [10]. A recent study suggests that CTPA use has increased, while yield has decreased, in metropolitan hospitals in Western Australia [15], highlighting potential overuse. Similarly, a study undertaken in Queensland identified that CTPA was likely overused in over half of the study cohort [16]. To date, there appear to be no studies conducted in Tasmania or regional areas of Australia. Therefore, this study aimed to explore the use of evidence-based practice within regional Tasmanian emergency departments in relation to CTPA order by determining whether CTPAs are ordered in accordance with validated CPGs.

### Choosing Wisely

Choosing Wisely Australia is a healthcare safety and quality initiative that aims to support clinicians in making good decisions around the most beneficial tests, treatments, and procedures for their patients. The overall goal of Choosing Wisely is to promote dialogue around unnecessary diagnostic activities where evidence suggests little or no benefit or increased risk of harm to improve patient outcomes and healthcare system efficiencies [17].

## 2. Materials and Methods

### 2.1. Study Design

We conducted a retrospective medical record review of all patients who underwent CTPA across all public emergency departments in Tasmania between 1 August 2018 and 31 December 2019 inclusive. The study period was indicated by using a power calculation to determine the minimum number of participants required from each site, with a power of 90% and a significance set at 5%. Participants were identified through an electronic ordering system used by all emergency departments.

### 2.2. Data Collection

Clinical information was collected from the digital medical record (DMR) and inputted into an Excel spreadsheet. Data collected included demographic data, presenting complaint(s), triage score, the clinical workup before CTPA, and the CTPA results. As part of the clinical workup, laboratory results for D-dimer were collected if available. The hospitals’ laboratory reference range was used, which for D-dimer was <0.5 mg/L. The formal radiology report was used as the definitive scan result and recorded as positive, negative, or equivocal. The PE type was recorded as the highest-level vessel with an embolus, as described in the formal radiology report. The yield was defined as the number of CTPA reports with any acute PE present as a proportion of all CTPAs performed. Alternative diagnoses other than PE explained the patients’ presenting symptoms.

In contrast, incidental findings were defined as a previously undiagnosed condition identified unintentionally during the scan. In cases of dual pathology, where both a PE and an alternative diagnosis and/or an incidental finding were identified, both results were recorded and included for analysis. The triage score was recorded according to the Australasian Triage Scale (ATS) Categories 1–5. Local emergency departments used ATS scores outside of 1–5 to identify patients not initially treated within the emergency department, and these were therefore excluded. In addition, the use of CPGs, as documented in the clinical notes, was recorded.

Regarding the Wells Criteria for PE, which can be stratified using either a two-tiered (PE unlikely or PE likely) or a three-tiered model (low, moderate, or high risk), if not specified by the clinician, we chose to stratify patients by the two-tiered model, as it meant there was a lower threshold for ordering a CTPA. Avoidable imaging was defined as CTPAs conducted against recognised clinical practice guidelines (in low-risk patients with a negative D-dimer). Potentially avoidable imaging was defined as CTPAs conducted despite an incomplete assessment, in patients with a negative D-dimer and no documented CPG used or in patients with a low pre-test probability of PE but no D-dimer conducted.

### 2.3. Data Analysis

Results were analysed using IBM SPSS Statistics Version 28 (IBM Technology Corp, New York, NY, USA). The chi-square test for goodness of fit was used to investigate the difference between scan results and the ‘ideal’ yield of 15%, as specified in the literature. The chi-square test for independence was utilised to determine relationships between categorical values, including hospital, gender, scan results, CPG use and results, D-Dimer use and results, age-adjusted D-Dimer results and clinician level. When Chi-square assumptions were violated (less than five observations per cell), a Monte Carlo test was utilised to determine the difference in yield based on CPG results and clinician level. A one-way analysis of variance (ANOVA) test was included to investigate significant differences in means for categorical versus numerical variables, including LOS and D-dimer utilisation. Finally, the independent-sample *t*-test was utilised to determine the age difference based on scan results.

### 2.4. Ethics

Ethics approval was obtained from the University of Tasmania’s Human Research Ethics Committee (HREC reference number: H0023434).

## 3. Results

### 3.1. Participant and Site Demographics/Characteristics

The total number of CTPAs ordered from emergency departments across the four sites during the study period was 2957. One-hundred and ninety-nine records were excluded: 186 as the scan was ordered but not completed; 12 due to a triage score outside of the ATS Categories 1–5; and 1 due to unavailable records. The total number of CTPAs included in this study was 2758 across Hospitals A (36.6%, *n* = 1009), B (15.2%, *n* = 419), C (26.5%, *n* = 730) and D (21.8%, *n* = 600) (Table 1). The mean age of the participants was 60.58 years (range: 16 to 100 years), and 1588 (57.6%) of the patients were female. No statistically significant differences were found in age or gender distribution between the four hospitals. There was a statistically significant difference in mean age between those patients with positive (*M* = 63.29) versus a negative (*M* = 60.29) scan result (*p* = 0.002). Males had a higher incidence of scans positive for PE than females. The most common presenting complaints for patients who underwent CTPA were shortness of breath, chest pain and palpitations/tachycardia, consistent across the four hospitals.

The overall yield for all CTPA performed across the sites was 12.4%, ranging from 8.2% to 16.1% (*p* = 0.002) (Table 2). A chi-square goodness-of-fit test indicates there was a significant difference in the proportion of positive CTPAs identified overall in our study (12.4%), as compared with the value of 15% suggested being the minimum yield, *X*^2^ (2, *n* = 2758) = 14.227, *p* ≤ 0.001). The number of CTPAs conducted per 1000 emergency department presentations during the study period was mostly consistent across Hospitals A, B and C, with Hospital D ordering, on average, 3.4 more CTPAs per 1000 presentations than the other three hospitals (Table 2). Alternative diagnoses were identified in 23.1% of CTPAs conducted across the study period, with the most common diagnoses being pneumonia (7.6%, *n* = 209) and pleural effusion (6.1%, *n* = 168). The most common incidental finding on CTPA was pulmonary nodules (6.0%, *n* = 166).

There were statistically significant associations between scan results and D-dimer laboratory results, *X*^2^ (4, *n* = 2758) = 0.050, *p* = 0.008), as well as scan results and age-adjusted D-dimer results, *X*^2^ (4, *n* = 2758) = 0.084, *p* ≤ 0.001). No statistically significant association was found between scan results and CPG or D-dimer utilisation. There was a difference in CTPA yield based on CPG results (indicated by the Monte Carlo test where Fisher-Freeman-Halton Exact Test Value 13.451; Monte Carlo Sig (2-sided) *p* = 0.026 (based on 10,000 sampled tables with starting seed 624,387,341); 99% CI: 0.022 (lower bound), 0.030 (upper bound)).

There was significant variation in the length of stay in the emergency department between the four hospitals (*p* ≤ 0.001). However, in those patients who were ultimately discharged from the emergency department (*n* = 1002), a one-way between-groups analysis of variance identified that there was a statistically significant increase in length of stay when a D-dimer was conducted before proceeding to CTPA (*p* = 0.024), from *M* = 340 to *M* = 361 min.

### 3.2. CPG Use and Adherence

There was significant variation in CPG documentation and D-dimer utilisation before scans across the four hospitals (*p* ≤ 0.001) (Table 3). Overall, 52.1% of participants had neither a CPG documented nor a D-dimer conducted before their scan. A CPG was documented before 11.8% of scans, while D-dimer was conducted before 43% of CTPAs. Over 7% of all scans conducted were completed on participants with a negative D-dimer result. A sub-analysis of participants aged 50 years and over was conducted. In this group of participants, 62.7% had neither a CPG nor a D-dimer conducted prior to proceeding to CTPA. For participants aged over 50 years, the age-adjusted D-dimer was also calculated. Of those over 50, 23% had a negative age-adjusted D-dimer before their scan. The number of avoidable scans conducted was eight, making up 0.3% of all CTPAs, while the number of potentially preventable scans totalled 231, over 8% of all scans conducted across the four hospitals.

A chi-square test for independence revealed a statistically significant association between clinician level and CPG use, *X*^2^ (4, *n* = 2758) = 0.099, *p* ≤ 0.001), where the more junior a clinician was, the more likely they were to document a CPG before conducting a CTPA (Table 4). However, no statistically significant differences were found between clinician levels for D-dimer utilisation, *X*^2^ (4, *n* = 2758) = 0.056, *p* = 0.068), nor was there a statistically significant difference in yield based on the ordering clinicians’ level of experience (indicated by Monte Carlo test where Fisher-Freeman-Halton Exact Test Value 10.368; Monte Carlo Sig (2-sided) *p* = 0.210 (based on 10,000 sampled tables with starting seed 2,000,000); 99% CI: 0.200 (lower bound), 0.220 (upper bound)).

## 4. Discussion

This study focused on CTPA utilisation across Tasmania’s four public emergency departments. In 2022, approximately 170,000 patients were seen across the four emergency departments, where presentations have been steadily increasing over the past decade, predominately due to population growth, an ageing population and increased incidence of chronic disease [18]. This study aimed to explore the use of evidence-based practice within regional Tasmanian emergency departments in relation to CTPA ordering. Our findings were consistent with the current literature, which found that CTPAs continue to be overused in emergency departments [14,19,20,21,22]. This study found significant variation in yield between the four study sites. Three of the four sites had a CTPA yield below the recommended 15% [4,11], with an overall yield of 12.4%, significantly below the target value. Very low CTPA yield (less than 10%) is rarely described in literature from outside the United States of America [4,10]; however, Hospital D yielded 8%. There are various potential explanations for this finding, which may include staffing considerations such as an increased number of locum medical officers who may not be familiar with the caseload or a lack of education on the use of or adherence to CPGs. The findings also highlight that the clinical pathway may differ at Hospital D, as overall, the participants’ primary complaint was chest pain, whereas, at the other three hospitals across the state, the primary complaint was shortness of breath. Alternative diagnoses were found in just over 23% of CTPAs, which does not align with the recommendations from the Royal College of Radiologists [11], which alongside their recommended yield of 15% for PE, state that 50% of scans should identify an alternative diagnosis. The findings do, however, align with current literature, where the rate of alternative diagnoses identified on CTPA falls between 10–35% [23,24,25].

Hospital D had a higher CTPA utilisation rate than the other three hospitals in this study, with 14.37 CTPAs conducted per 1000 emergency department presentations. This is also notably higher than the utilisation rate presented in other studies, as in Mountain, Keijzers [4]’s multi-site Australasian study, overall they found that 6.2 CTPAs were conducted for every 1000 presentations, and in Salehi, Phalpher [3]’s Canadian study they identified a utilisation rate of 9.96 per 1000 presentations. While it is understood that a very high utilisation rate is problematic, it is also important to note that on the other end of the spectrum, a very low utilisation rate could have implications for equipment maintenance and correct usage.

The use of D-dimer and age-adjusted D-dimer to rule out PE in low-risk patients is validated in literature and was supported in this study, where those with positive D-dimer results were more likely to have CTPAs positive for PE. In their research, Booker and Johnson [19] found 10% of CTPAs were ordered for patients with negative D-dimers. This was like our findings, where 90 (7.6%) participants underwent CTPA despite a negative D-dimer. Furthermore, almost a quarter of participants aged 50 years and older had a negative D-dimer when it was age-adjusted. This further highlights that using D-dimer to rule out PE is not completely accepted in the clinical setting [19]. Participants in Gyftopoulos, Smith [26]’s study raised concerns that completing a D-dimer instead of proceeding directly to CTPA would increase the length of stay. This was supported in this study, where the length of stay for participants who were ultimately discharged from the emergency department increased for those who had a D-dimer conducted before proceeding to CTPA. Despite reaching statistical significance, the actual difference in mean scores was just 21 min.

Senior clinicians are likelier to have a higher scan yield than junior clinicians [27,28]. However, this was not supported in the current study, where there was no significant difference in yield based on the level of experience of the ordering clinician. Chen and Gray [24] found similar results in their study, where CTPA utilisation and positivity rates did not correlate with the clinician’s experience level. While there were no significant differences between clinician levels for D-dimer utilisation, junior clinicians were more likely to document the use of a CPG than their senior counterparts. Rowlands, Tariq [29] highlighted that the more senior a clinician is, the amount and level of detail included in their documentation decreases. This study could be due to the junior clinicians’ recent medical education, which is likely to have emphasized the importance of adhering to clinical guidelines that support best practices across all areas, not just for investigating suspected PE.

Over 50% of patients presenting to Tasmanian emergency departments with suspected PE had neither a clinical practice guideline documented nor a D-dimer conducted before proceeding to CTPA, which may suggest that patients are being inappropriately screened for the existence of a PE. A sub-analysis of those aged 50 years and older was also conducted as the PE Rule Out Criteria does not apply to this age group. Therefore, at a minimum, they must have a D-dimer conducted before proceeding to CTPA. As a result, there may have been higher utilisation of CPGs and/or D-dimer testing in this cohort. However, in this group of participants, 62.7% had neither a CPG nor a D-dimer conducted prior to proceeding to CTPA. While it is difficult to quantify the rate of imaging that is truly ‘guideline-discordant’ in this study, predominately due to the limited documentation of CPG use, these figures are still substantial, especially compared to other published studies which identified much lower rates. For example, Simon, Miake-Lye [30] found the frequency of guideline-discordant ordering behaviour in their emergency department to be between 25–37%, and Al Dandan, Hassan [31] found that 18–33% of their scans ordered did not adhere to CPGs.

This indicates that CTPAs are being ordered against the Choosing Wisely recommendations within Tasmanian emergency departments. There are many potential reasons for this. However, most likely because the emergency departments are fast-paced, busy environments with flow-related demands and patients requiring time-critical care, there may be limited time for detailed documentation. Another potential explanation could be a lack of education regarding the use of CPGs. The literature highlights other reasons for overuse in general, including clinician and/or patient fear of missing a diagnosis, fear of malpractice and/or litigation, lack of clinician knowledge, time constraints [32] and increased CT availability within emergency departments [15], with these reasons potentially applicable in the Tasmanian setting also.

As outlined above, all four hospitals conducted potentially avoidable imaging by scanning patients with a negative D-dimer, or negative when age-adjusted, with a low-risk pre-test probability, or no documented risk-stratification score. The potentially preventable scans totalled 231, over 8% of all scans conducted across the four hospitals. This number of potentially avoidable scans is much lower than the rates reported in other studies, which range from 20–49.5% [22,33,34]. Three of the 231 patients went on to have a positive scan for PE, which is consistent with the false negative rates presented in the literature [6,35]. The number of avoidable scans was 8, 0.3% of all scans conducted. All 8 of the avoidable scans conducted were negative for PE.

This medical record review found significant variability in ordering practices concerning the adherence to and use of CPGs. Clinicians infrequently documented clinical practice guideline use before ordering a CTPA. Increasing the use of CPGs and D-dimer testing has been highlighted in the literature as the predominant strategy for reducing unnecessary CTPAs and increasing scan yield [14,36,37]. However, this strategy was not supported by the findings of this study, where no statistically significant association was found between scan yield and CPG or D-dimer utilisation within the participants who had either of these conducted as part of their pre-scan clinical workup. Therefore, understanding the clinicians’ rationale for ordering a CTPA within this clinical context is crucial to design future interventions aimed at reducing unnecessary medical imaging [30].

## 5. Limitations

There are several limitations to this study. First, this study relied on retrospective data collected from routine clinical practice. Second, the study cohort was only from public hospitals across Tasmania, meaning the results might not be generalisable to other settings. For example, in some clinical settings, D-dimers may be ordered without the guidance of a CPG as part of investigating PE. Third, each CTPA was reported on by one radiologist, and we did not check for variability between clinicians’ reports. However, these reports are relied upon for clinical diagnosis and management, so this may not present a significant concern. Further qualitative research is required to identify explanations for our findings. Following this study, the authors will conduct semi-structured interviews with clinicians working within Tasmanian emergency departments to develop a deeper understanding of the emerging themes, especially regarding ordering behaviour.

## 6. Conclusions

Tasmanian emergency departments are not consistently ‘Choosing Wisely’ when investigating PE, with CTPAs continuing to be overused. Whilst specific reasons for overuse are still being investigated, this study demonstrates a need for targeted action in ensuring that CPTAs are ordered appropriately by adhering to the guidelines. In current clinical practice, CPGs and D-dimer are often underused, leading to significant losses in the healthcare system due to the overuse of expensive diagnostics.

## Figures and Tables

**Table 1:** Participant demographics and characteristics.

		Hospital A *n* = 1009 (36.6%)	Hospital B *n* = 419 (15.2%)	Hospital C *n* = 730 (26.5%)	Hospital D *n* = 600 (21.8%)	Total *n* = 2758
**Age (mean)**		59.92	60.68	61.00	61.12	60.58
**Gender**						
	Male	424 (42.0%)	171 (40.8%)	330 (45.2%)	245 (40.8%)	1170 (42.4%)
	Female	585 (58.0%)	248 (59.2%)	400 (54.8%)	355 (59.2%)	1588 (57.6%)
**Presenting Complaint**						
	SOB	66.8%	65.2%	64.5%	62.0%	69.4%
	Chest Pain	60.3%	58.7%	63.8%	68.0%	67.0%
	Palpitations/Tachycardia	33.1%	31.5%	32.3%	30.3%	34.3%
	Other	37.8%	31.0%	31.5%	25.5%	34.7%

**Table 2:** Site Characteristics.

	Hospital A N = 1009	Hospital B N = 419	Hospital C N = 730	Hospital D N = 600	Total N = 2758
**ED attendance during the study period**	90,564	39,617	64,857	41,750	236,788
**Scans positive for PE (yield)**	162 (16.1%)	49 (11.7%)	83 (11.4%)	49 (8.2%)	343 (12.4%)
**CTPAs conducted/1000 ED attendances**	11.14	10.58	11.26	14.37	11.65
**Yield/1000 ED attendances**	1.79	1.24	1.28	1.17	1.45
**Length of stay (mins) in ED (mean)**	590.48	492.50	823.77	466.99	606.46

**Table 3:** CPG adherence.

		Hospital A *n* = 1009	Hospital B *n* = 419	Hospital C *n* = 730	Hospital D *n* = 600	Total *n* = 2758
**Clinical practice guidelines documented before the scan**		176 (17.4%)	30 (7.2%)	73 (10.0%)	47 (7.8%)	326 (11.8%)
**D-dimer used before the scan**		538 (53.3%)	144 (34.4%)	267 (36.6%)	237 (39.5%)	1186 (43.0%)
**D-dimer result**						
	Positive	510 (94.8%)	129 (89.6%)	243 (91.0%)	213 (90.3%)	1095 (92.4%)
	Negative	28 (5.2%)	15 (10.4%)	24 (9.0%)	23 (9.7%)	90 (7.6%)
**Age-adjusted D-dimer result (for >50 years)**						
	Positive	297 (83.2%)	76 (80.0%)	132 (71.7%)	107 (67.3%)	612 (77.0%)
	Negative	60 (16.8%)	19 (20.0%)	52 (28.3%)	52 (32.7%)	183 (23.0%)

**Table 4:** Variation between senior and junior clinicians.

Clinician Level	Consultant	Registrar	Resident	Intern	Unknown
**CTPAs ordered + conducted**	424 (15.4%)	1366 (49.5%)	511 (18.5%)	423 (15.3%)	34 (1.2%)
**Yield (% of all scans ordered by that clinician level)**	56 (13.2%)	172 (12.6%)	70 (13.7%)	41 (9.7%)	4 (11.8%)
**Clinical practice guidelines documented before the scan**	29 (6.8%)	149 (10.9%)	69 (13.5%)	75 (17.7%)	5 (14.7%)
**D-dimer used before the scan**	186 (43.9%)	613 (44.9%)	202 (39.5%)	176 (41.6%)	9 (26.5%)

## Data Availability

The data presented in this study are available on request from the corresponding author. The data are not publicly available due to ethical restrictions.
